# *Gemella* Species Bacteremia and Stroke in an Elderly Patient with Respiratory Tract Infection

**DOI:** 10.1155/2017/1098527

**Published:** 2017-01-02

**Authors:** Sriraksha Jayananda, Narasimha Swamy Gollol-Raju, Nada Fadul

**Affiliations:** Vidant Medical Center, East Carolina University, 600 Moye Blvd., Greenville, NC 27834, USA

## Abstract

*Gemella* species are part of normal human flora. They are rarely associated with infections. As opportunistic pathogens, they can cause life-threatening infection in individuals with risk factors. We present an unusual case of an elderly patient, with no predisposing risk factors, who presented with respiratory tract infection and* Gemella *species bacteremia and suffered a stroke in the absence of features of endocarditis.

## 1. Introduction

*Gemella* species (GS) are part of the normal flora of human mucous membranes. First reported in 1917, GS are facultative anaerobic, catalase-negative, gram-positive cocci. GS are found in pairs, clusters, or chains. They have a typical gram-positive cell wall structure but they easily decolorize during the gram staining process rendering them gram variable. These characteristics make them difficult to identify with standard laboratory techniques and as such the actual prevalence of infection may be underestimated [1, 2]. As opportunistic pathogens, GS can cause life-threatening infections in individuals with predisposing risk factors. We hereby report a case of an elderly gentleman with no predisposing risk factors for GS infection who presented with lower respiratory tract infection and developed GS bacteremia with subsequent dense hemiplegia with absent endocarditis features.

## 2. Case Report

An 82-year-old Caucasian male with no significant past medical history presented with several days of fever, malaise, minimally productive cough, and some confusion. He was febrile with a rectal temperature of 40.4°C, heart rate of 143/minute, blood pressure of 154/70 mmHg, and respiratory rate of 20/minute with oxygen saturation of 98% in room air. His physical examination revealed good dental hygiene, no mucocutaneous lesions, few basal crackles on lung auscultation, and no cardiac murmurs. He denied any recent dental, genitourinary, or gastrointestinal procedures. He was not on any immunosuppressant medications. His white blood cell count was 15,100/*μ*L with 91.2% neutrophils. Electrocardiogram showed sinus tachycardia. Chest radiogram showed clear lung fields, and a noncontrast head computed tomography (CT) scan showed age related changes with no acute process. Patient met the systemic inflammatory response syndrome (SIRS) criteria and possible lower respiratory tract infection. Two sets of blood cultures were drawn and sputum was sent for gram staining and culture. Patient was started on ceftriaxone and azithromycin. Sputum culture showed* Haemophilus influenzae* beta-lactamase positive growth. By day three of hospitalization, blood cultures became positive for gram-positive cocci considered to be* Staphylococcus* species. Blood cultures were repeated and vancomycin was added to the antibiotic regimen. Patient had shown clinical and laboratory improvement with the above regimen with resolution of SIRS criteria. By the fifth day, the organism was identified as* Gemella* species (our laboratory was unable to further identify the organism) and later that day patient developed dense left hemiplegia and facial palsy. A noncontrast head CT scan showed no acute areas of infarct. A transthoracic echocardiogram was negative for vegetation or any significant structural or valvular abnormality. Clinical evidence of endocarditis was not appreciated and the patient did not meet modified Duke criteria for endocarditis or possible endocarditis. Etiology of the stroke was considered likely primary central nervous system vascular event, and as such routine acute ischemic stroke management was initiated. Unfortunately, shortly after the devastating stroke, the patient passed away from complications related to his initial stroke management and hence further imaging studies could not be performed. Family declined autopsy. The repeat set of blood cultures drawn on day three of hospitalization remained negative.

## 3. Discussion

*Gemella* species are an unusual cause of human infections. However, as opportunistic pathogens, they are known to cause serious and fatal infections in both pediatric and adult population with risk factors [3–5]. Predisposing risk factors are underlying heart disease (valvular disease, hypertrophic cardiomyopathy, myxoma, congenital heart disease, and prosthetic valves), poor dental hygiene, diabetes mellitus, intravenous drug use, steroid therapy, hepatorenal syndrome, and dental and gastrointestinal procedures [1, 4, 6]. The majority of cases reported are of endocarditis. Additionally, cases of central nervous system infections, embolic stroke from underlying endocarditis, lung abscess, peritonitis in dialysis dependent patients, septic arthritis, and sepsis have also been reported [2–9]. Confirmation of infection is by bacterial isolation and performing phenotypic observation for morphology.* Gemella* should be of consideration, especially in high risk individuals, with endovascular infections when standard methods identify slow growing catalase-negative gram-positive cocci. Newer technique, 16S rRNA gene analysis, may be more efficient and accurate in the identification of* Gemella* spp. [1, 2, 7]. Most cases of infections are successfully treated with antimicrobial therapy alone. Penicillin in combination with gentamicin, and in penicillin allergic or resistant cases, vancomycin monotherapy, or combination of erythromycin and rifampin had been successful [2, 4, 5]. Early surgical intervention is needed where appropriate.

## 4. Conclusion

*Gemella* species, as opportunistic pathogens, can cause life-threatening infections in individuals with predisposing risk factors. As noted in our case, usual risk factors may not be always present but advanced age should be considered a predisposing risk factor for* Gemella* species infection. Primary respiratory tract infection and infections or disruption of the gastrointestinal and genitourinary tract mucosa could lead to invasion and secondary infection with* Gemella* species as they are normal flora of these tracts. As such, in these circumstances,* Gemella* species infection should be of consideration when culture media demonstrate slow growing gram-positive organism. Our case is unusual in that* Gemella* species bacteremia was noted without any endovascular or other organ involvement that could be identified. The cause of our patient's stroke is likely related to primary central nervous system vascular disease but the possibility of septic emboli related to* Gemella* species endocarditis could not be entirely ruled out.
